# Immune cell landscapes are associated with high-grade serous ovarian cancer survival

**DOI:** 10.1038/s41598-024-67213-4

**Published:** 2024-07-12

**Authors:** Guoan Zhang, Yan Zhang, Jingjing Zhang, Xiaohui Yang, Wenjie Sun, Ying Liu, Yingfu Liu

**Affiliations:** 1 Science and Technology Experiment Center, Cangzhou Medical College, Cangzhou, 061001 People’s Republic of China; 2 Cangzhou Nanobody Technology Innovation Center, Cangzhou Medical College, Cangzhou, 061001 People’s Republic of China; 3 University Nanobody Application Technology Research and Development Center of Hebei Provice, Cangzhou, 061001 People’s Republic of China

**Keywords:** High-grade serous ovarian cancer, T cell activation, Overall survival, Macrophage, Cell ratios, Immunology, Cancer microenvironment

## Abstract

High-grade serous ovarian cancer (HGSOC) is an aggressive disease known to develop resistance to chemotherapy. We investigated the prognostic significance of tumor cell states and potential mechanisms underlying chemotherapy resistance in HGSOC. Transcriptome deconvolution was performed to address cellular heterogeneity. Kaplan–Meier survival curves were plotted to illustrate the outcomes of patients with varying cellular abundances. The association between gene expression and chemotherapy response was tested. After adjusting for surgery status and grading, several cell states exhibited a significant correlation with patient survival. Cell states can organize into carcinoma ecotypes (CE). CE9 and CE10 were proinflammatory, characterized by higher immunoreactivity, and were associated with favorable survival outcomes. Ratios of cell states and ecotypes had better prognostic abilities than a single cell state or ecotype. A total of 1265 differentially expressed genes were identified between samples with high and low levels of C9 or CE10. These genes were partitioned into three co-expressed modules, which were associated with tumor cells and immune cells. *Pogz* was identified to be linked with immune cell genes and the chemotherapy response of paclitaxel. Collectively, the survival of HGSOC patients is correlated with specific cell states and ecotypes.

## Introduction

Ovarian cancer is one of the most deadly gynecologic malignancies^1^. Ovarian low-grade serous carcinomas (LGSOC) have been identified as a separate disease entity from more common high-grade serous ovarian cancer (HGSOC). HGSOCs are characterized by aggressive behavior, advanced stage, and genetic instability^2^. The common treatments for HGSOC are surgery, chemotherapy, and immunotherapy. However, HGSOC patients' overall survival (OS) has not improved much in the last three decades^3^.

Immune dysregulation is a hallmark in cancers including HGSOC^4^. Natural killer (NK) cells are components of innate immune defense and are important effectors in the first line of defense against transformed cells^5^. The ability of NK cells to kill tumors depends on the balance between stimulatory and inhibitory signaling by cytokines such as interferons and IL-2^6^. In the tumor microenvironment, TGFβ, IL-10, and IL-6 could suppress NK cell activity^7^, while IL-2, IL-12, and type I interferons could positively regulate NK cell function^5^. The presence of immune-mediated survival benefits in HGSOC has been proved by estimating immune cell type proportions within HGSOC tumors^8^. The correlation between immune infiltration and survival following platinum-based chemotherapy in HGSOC has been evaluated^3^. The study revealed an elevated CD163/CD68 ratio (indicative of M2 macrophage proportion) in HGSOC, suggesting the unfavorable impact of M2 macrophages in the context of HGSOC^1^. This finding was also consistent with another study's findings, which found that high infiltration level of M2 macrophages was associated with poor outcomes^9^.

Flow cytometry and immunohistochemistry, have been used to study cellular heterogeneity, but they are limited by the availability of specific antibodies and the number of cell types that can be simultaneously assessed. Single-cell RNA sequencing (scRNA-seq) is a powerful strategy for resolving cellular heterogeneity at high resolution without prior knowledge^10^. However, scRNA-seq may not be practical for large-scale analyses and is expensive. Digital cytometry techniques can infer cell-type-specific gene expression profiles without physical cell isolation and are becoming complementary to scRNA-seq^11^. CIBERSORTx allows researchers to obtain estimates of cell type abundances in mixed cell populations and impute gene expression profiles, offering a comprehensive view of the cellular landscape in normal and disease states^12^. Ecotyper is based on CIBERSORTx but has advantages, such as the ability to identify cell states, quantify their relative abundance in each sample, recover them in external expression datasets, and discover co-association patterns between cell states that form the cancer ecosystems^11,13^. Multiomic approaches have been used to study the complex interactions between cells of the immune system and cancer^14^. Ecotyper enables the characterization of cellular heterogeneity within tumor microenvironments and provides insights into the interactions between various microenvironmental cells and malignant cells, contributing to our understanding of tumor progression and therapeutic resistance^13^.

**Figure 1:**
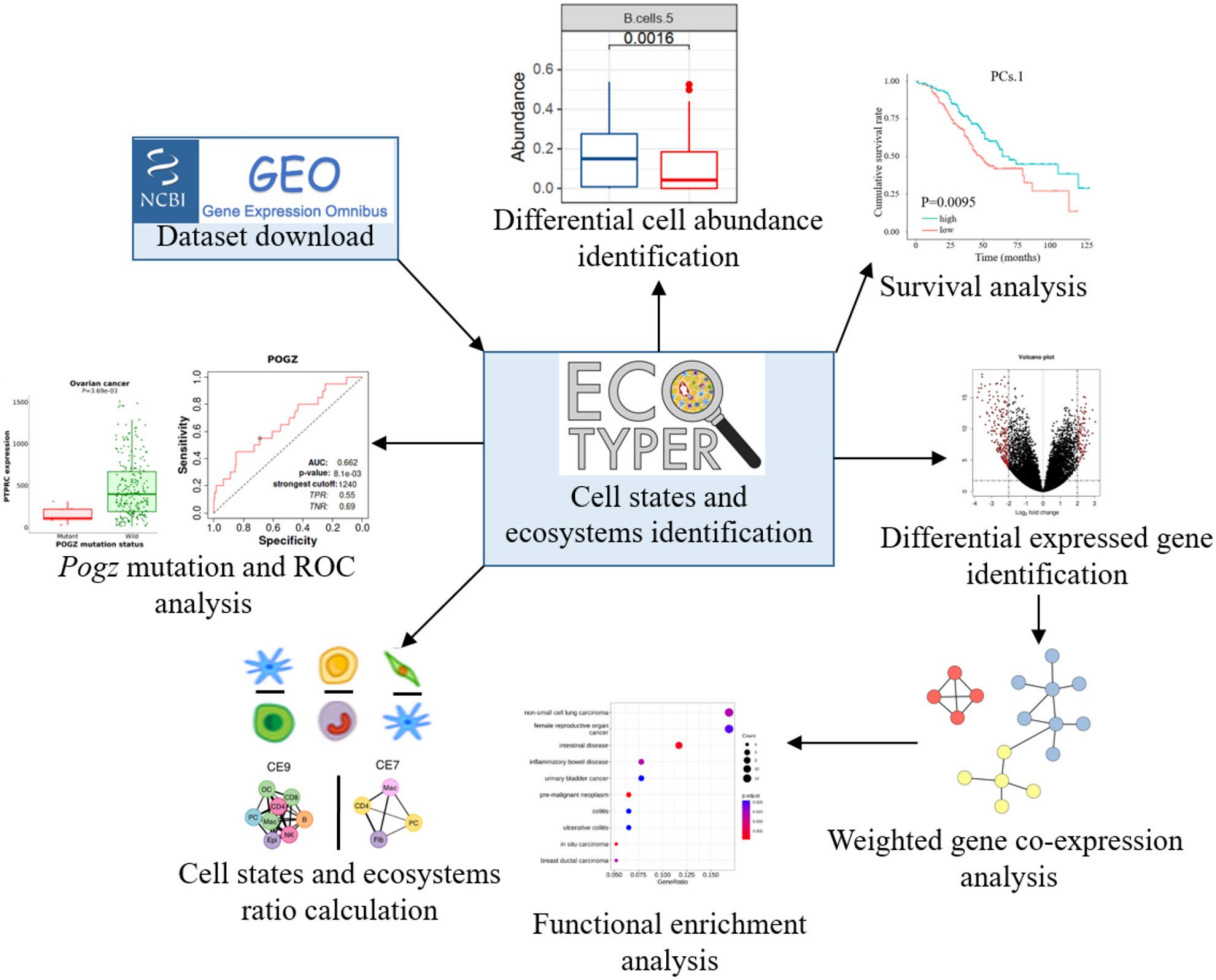
Schematic workflow of our study. The high-grade serous ovarian cancer (HGSOC) dataset was downloaded from the NCBI GEO database. The expression matrix was transformed into a cell states matrix and ecosystems matrix by Ecotyper. Then, the differential cell abundance between dead and alive patients was identified. Survival analysis based on cell states or carcinoma ecotypes was performed. Differentially expressed genes (DEGs) between two patients split by the survival time were identified. Weighted gene co-expression analysis (WGCNA) based on DEGs was executed. WGCNA modules were annotated by functional enrichment analysis. Ratios of cell states and carcinoma ecotypes were calculated for ratio-based survival analysis. Finally, potential mechanisms of therapeutic benefit with paclitaxel were inferred.

Currently, only a few studies used the traditional CIBSERSORTx to investigate the tumor microenvironment (TME) in HGSOC^3,8,9,15,16^. The cell states, cell ratios, and ecotypes have been studied across tumor types but in HGSOC they have not been comprehensively investigated^13^. Therefore, we aim to analyze the prognostic value of cell states, cell ratios, and ecotypes in HGSOC survival and chemotherapy response. We, for the first time, identified cell states, cell ratios, and ecotypes in HGSOC survival and chemotherapy response that merit further validation. The analysis workflow of the study is shown in Fig. 1.

## Materials and methods

### Dataset preparation

HGSOC microarray and survival data were downloaded from the NCBI GEO database at https://www.ncbi.nlm.nih.gov/geo/query/acc.cgi?acc=GSE32062^17^. The dataset was from a Japanese cohort, including 260 patients with ages of 58.2 ± 10.8. 204 and 56 patients were at stages III and IV, respectively. All the patients received the same chemotherapy with platinum and taxane. 103 and 157 patients were optimal and suboptimal in surgery status. The expression matrix with gene symbols as the first column was uploaded to the online Ecotyper tool at https://ecotyper.stanford.edu/carcinoma/^13^. Ecotyper is a powerful tool for transcriptomic analysis, particularly in the context of cell type-specific transcriptional states and cellular ecosystems, providing valuable insights into the complex composition of heterogeneous biological samples^13^. Ecotyper is a machine-learning framework for digital cytometry to identify cell-type-specific transcriptional programs (cell states), quantify their relative abundance in each sample, and determine the co-assignment of cell states into multicellular communities (ecotypes) from transcriptome data. The reference of cell states and ecosystems has been established across 16 types of human solid tumors^13^. EcoTyper performs the following modular steps, in silico purification, cell state discovery, ecotype discovery, and cell state and ecotype recovery. The resulting files for cell states and ecotypes were downloaded and merged into matrix files for downstream analysis. *Pogz* gene expression data were extracted from the TCGA and GTEx datasets by TNMplot^18–20^.

### Survival analysis

The relationships between cell states, ecotypes, ratios, or gene expression and patient overall survival (OS) were analyzed using multivariate Cox proportional hazards regression models within the R survival package. Association analyses were adjusted for the patient’s disease stage and surgery status. The patients were divided into two groups split at the median values of independent variables, including cell states, ecotypes, cell state ratios, and ecotype ratios. Kaplan–Meier survival curves for cell states and ecotypes were plotted by ggsurvplot in the R survminer package. The significance of the survival rate difference between the two groups was assessed using the log-rank test. Unless otherwise stated, a P value of less than 0.05 was considered significant. Survival curves for genes were plotted by Kaplan–Meier Plotter^21^. ROC curves were plotted by ROCplot.org^22^.

### Differential gene expression analysis and gene co-expression network construction

The differential gene expression in CE9- or CE10-high samples and other samples were analyzed by the R limma package. CE9- or CE10-high samples were identified from the result of ecotype assignment where the column of “carcinoma ecotype” was labeled “CE9” or “CE10”. In limma, they were labeled “1” as the experiment group, and other samples were labeled “0” as the control group. The intensity values were log_2_ transformed before differential gene identification by the eBayes method. The significant thresholds for log_2_(fold change) and Benjamini–Hochberg adjusted P values were set as 0.6 and 0.05. For gene co-expression module identification, the differential expression genes matrix was analyzed according to manuals^23,24^. The parameters were set as softPower = 12, corFnc = "cor", Networktype = "signed", minModuleSize = 20, deepSplit = 4. For module functional annotation, clusterProfiler was used to get the significant functional terms and related bubble plots^25^.

### Statistical analysis

The statistical significance of the mean difference between the two groups was calculated by the t test. The mean difference between multiple groups was calculated by the analysis of variance. In all statistical analyses, a P value of less than 0.05 was considered significant.

## Results

### Transcriptome-based cell states discovery and their prognostic associations in HGSOC

**Figure 2:**
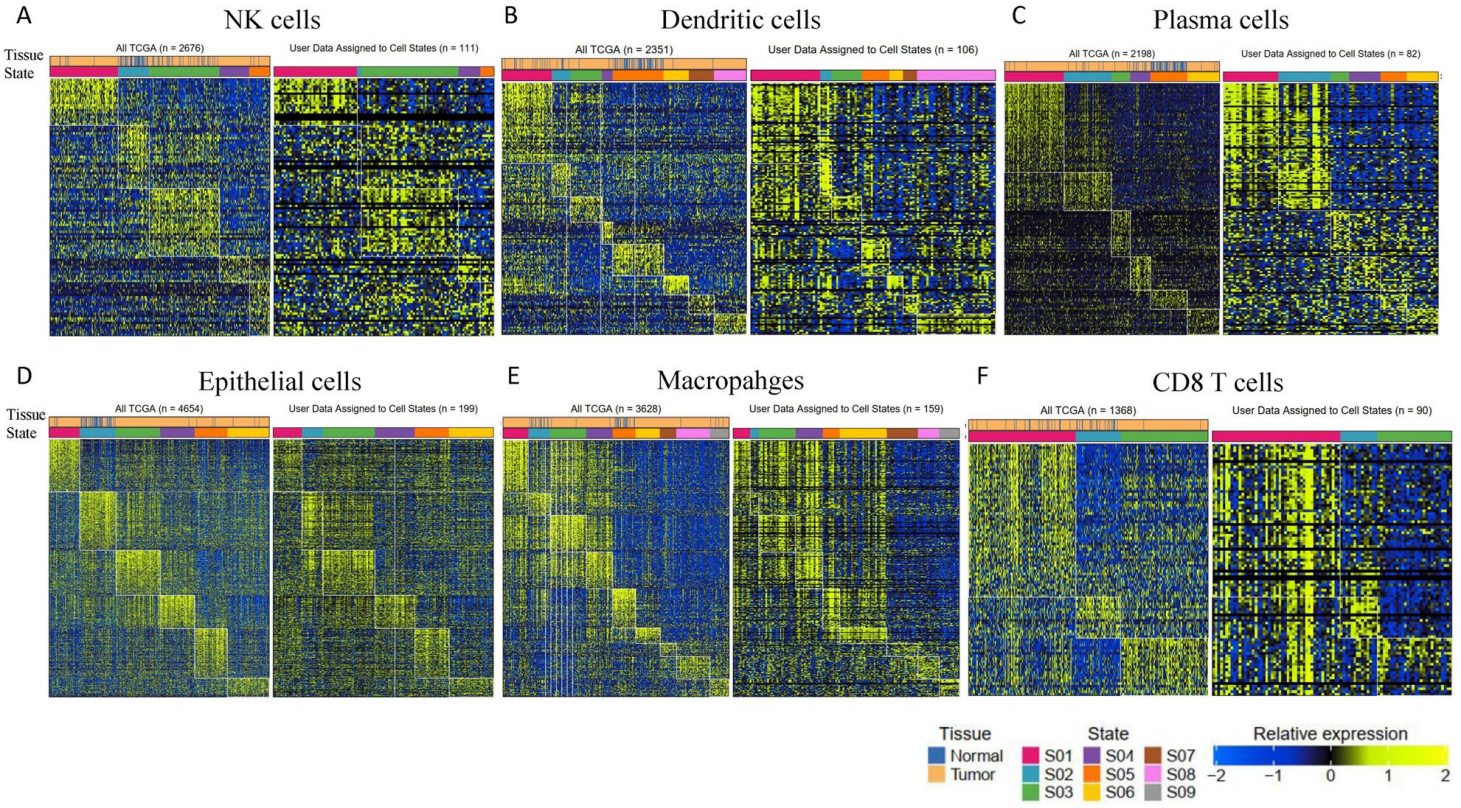
Heatmap depicting 5, 8, 6, 6, 9, and 3 cell states identified from digitally purified (**A**) natural killer cells (NKs), (**B**) dendritic cells (DCs), (**C**) plasma cells (PCs), (**D**) epithelial cells (ECs), (**E**) monocytes/macrophages (MCs), and (**F**) CD8 T (CD8T) cells, respectively. For each cell type, the left panel represents the cell states of the TCGA dataset, while the right panel represents the cell states of the current HGSOC dataset. Patient samples (columns) are organized by the most prevalent cell state and annotated with bulk tumor cell-of-origin labels.

We applied Ecotyper to HGSOC and found that cell states and ecotypes discovered in the TCGA cohort were reproducible in the HGSOC dataset. As an example, the following numbers of cell states were identified in various cell types: 5 in natural killer (NK) cells, 8 in dendritic cells (DCs), 6 in plasma cells (PCs), 6 in epithelial cells (ECs), 9 in monocytes/macrophages (MCs), and 3 in CD8 T cells (CD8T). (Fig. 2A–F). All the cell states, abundance, and assignment data were provided in Tables S1, S2.

**Figure 3:**
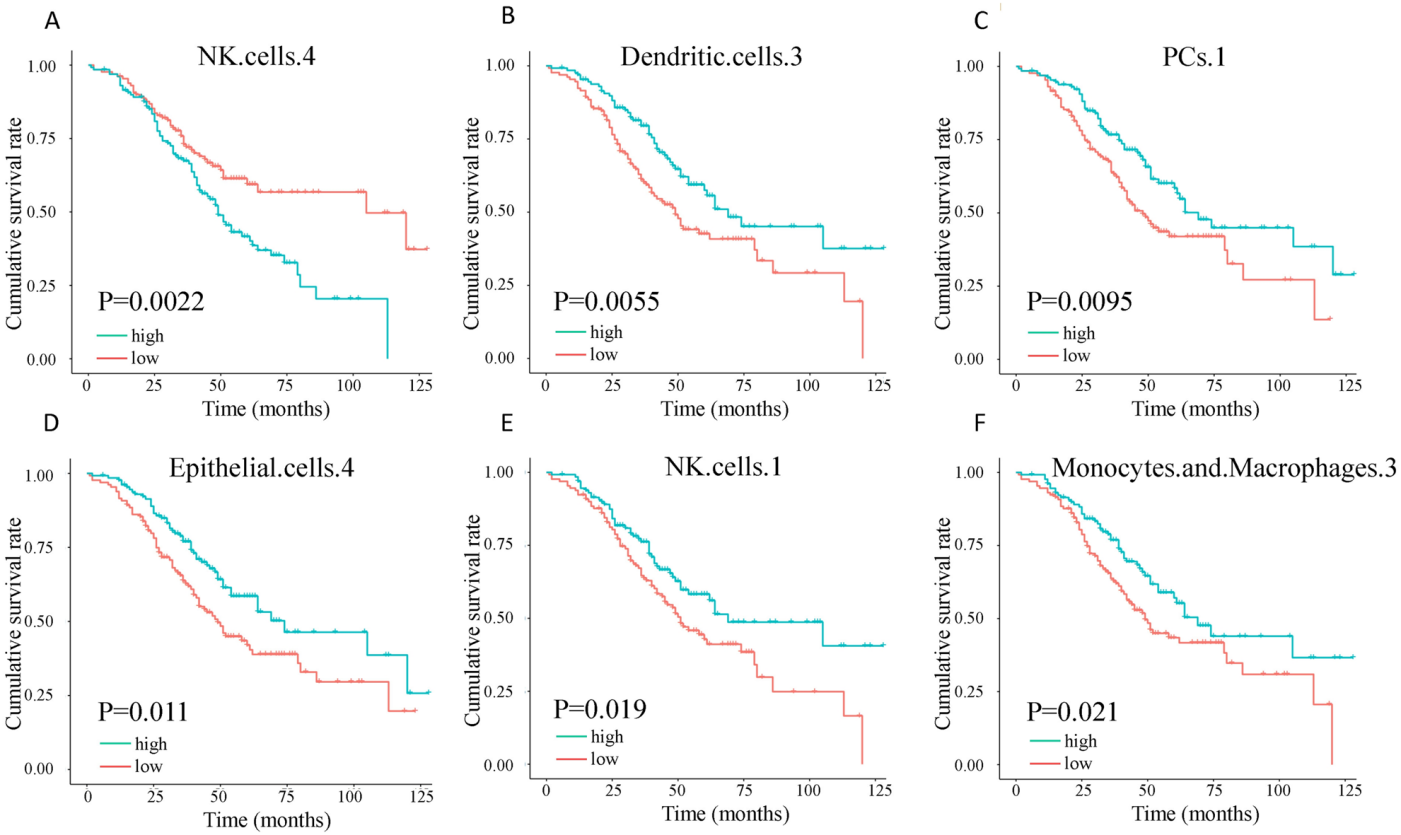
Overall survival (OS) for cell states (**A**) natural killer (NK) cells S4, (**B**) dendritic cells (DCs) S3, (**C**) plasma cells (PCs) S1, (**D**) epithelial cells (ECs) S4, (**E**) NK cells S1, and (**F**) monocytes/macrophages (MCs) S3 in the 260 HGSOC. Significance was determined by a two-sided log-rank test. The unit of time is the month.

The prognostic influence of cell states in HGSOC has not been comprehensively investigated. In this study, we utilized the abundance matrix of cell states to quantify their relationships with patient survival. We found that 13 of the 71 cell states were significantly associated with overall survival (Fig. 3A–F shows the top 6 cell states). All the cell states were significant in multivariable analysis incorporating surgery status and grading (Table 1). NKs S4 had the most significant prognostic ability, which was associated with an increased risk of death, while NKs S1 had a favorable prognosis (Fig. 3A,E). According to Ecotyper, NKs S4 is annotated as an unknown cell state. DCs S3 is annotated as mature immunogenic. PCs S1 is annotated as classical. ECs S4 is annotated as pro-inflammatory. NKs S1 is annotated as classical. MCs S3 is annotated as classical M1. A global survey of the 12 cell types (Table S2) with 71 cell states (Table S1) into favorable and adverse states suggested their composition and function heterogeneity. For these prognostic cell states, differential cell abundance was observed between survival and non-survival patients (Fig. S1).

**Table 1:** Multivariable analysis for the top 6 cell states and covariates.

	Variate HR (P)	Covariate HR (P)	
		Surgery_suboptimal	Grading
NK.cells.4	1.95 (5.0E−4)	2.18 (1.0E−4)	1.05 (0.79)
Dendritic.cells.3	0.56 (0.002)	2.11 (2.0E−4)	1.06 (0.74)
PCs.1	0.60 (0.008)	2.04 (4.0E−4)	1.07 (0.70)
Epithelial.cells.4	0.57 (0.003)	2.14 (1.0E−4)	1.09 (0.64)
NK.cells.1	0.65 (0.02)	2.01 (4.0E−4)	1.00 (0.99)
Monocytes.and.Macrophages.3	0.62 (0.01)	2.05 (3.0E−4)	1.08 (0.68)

### Mapping of multicellular communities to reference in HGSOC

**Figure 4:**
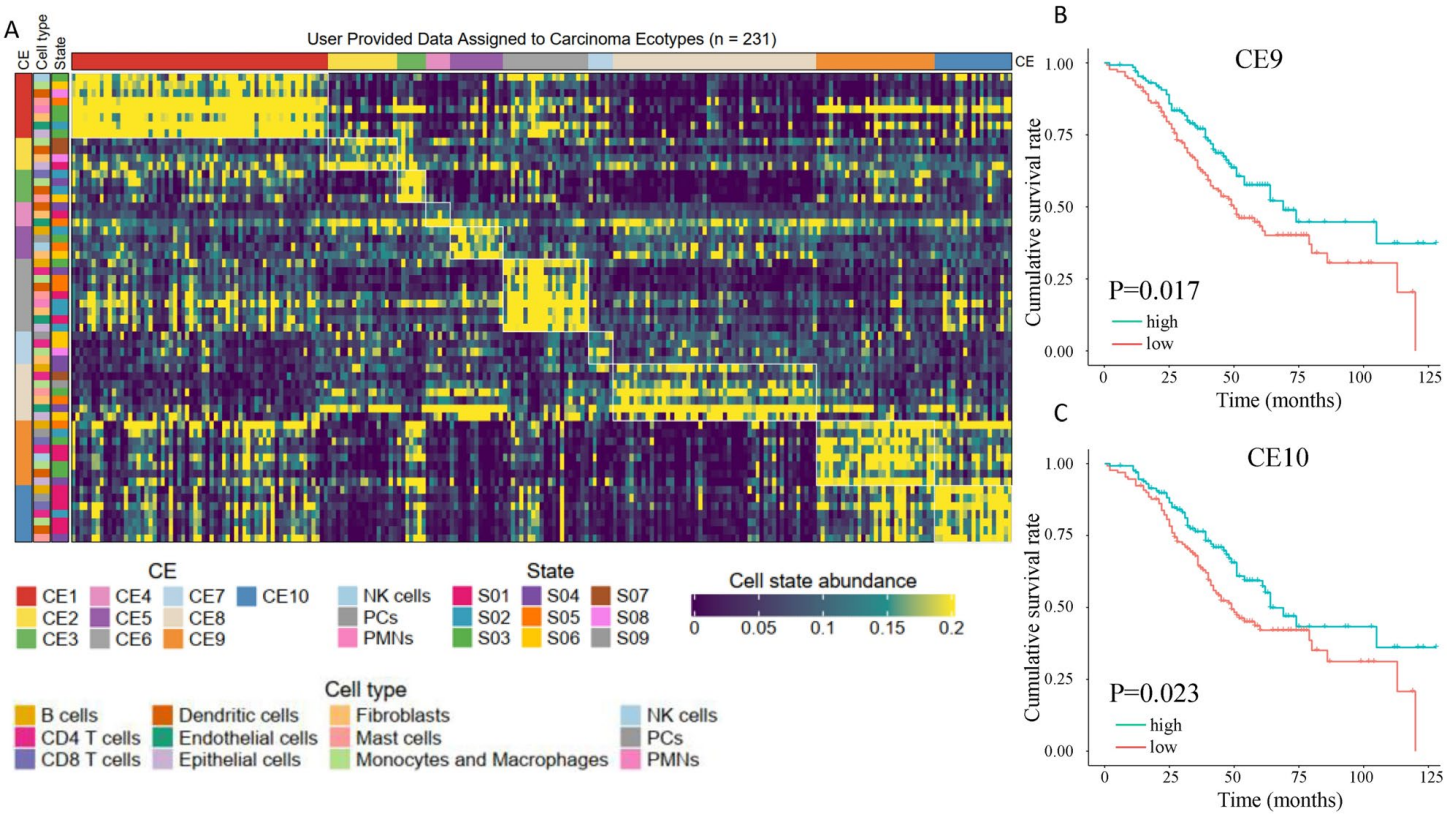
Ecotypes in HGSOC. (**A**) Cell-state abundance profiles are organized into ten carcinoma ecotypes (CEs). Only cell states and HGSOC samples assigned to CEs are shown. HGSOC samples are ordered by the most abundant CE class per specimen. (**B**) CE9 and (**C**) CE10 are associated with patient survival in the HGSOC dataset GSE32062. The unit of time is the month.

Tumors are complex ecosystems composed of interacting cells with distinct cell states and spatial distributions. EcoTyper identifies co-association patterns among different cell states and carcinoma ecotype (CE) is generally defined as a set of cell states that tend to co-occur in various independent tissue samples^13^. Ten CEs were identified (Fig. 4A). Among the assigned samples, the CE1 ecotype had the largest number of samples. CE1 is enriched with cancer hallmark pathway epithelial-mesenchymal transition (EMT). When associated with survival, we found that CE9 and CE10 were the two significant ecotypes positively correlated with survival after adjusting for surgery status and grading (Fig. 4B,C). All the cell ecotypes, abundance, and assignment data were provided in Tables S3, S4.

### Differential gene expression analysis in ecotypes CE9 and CE10

**Figure 5:**
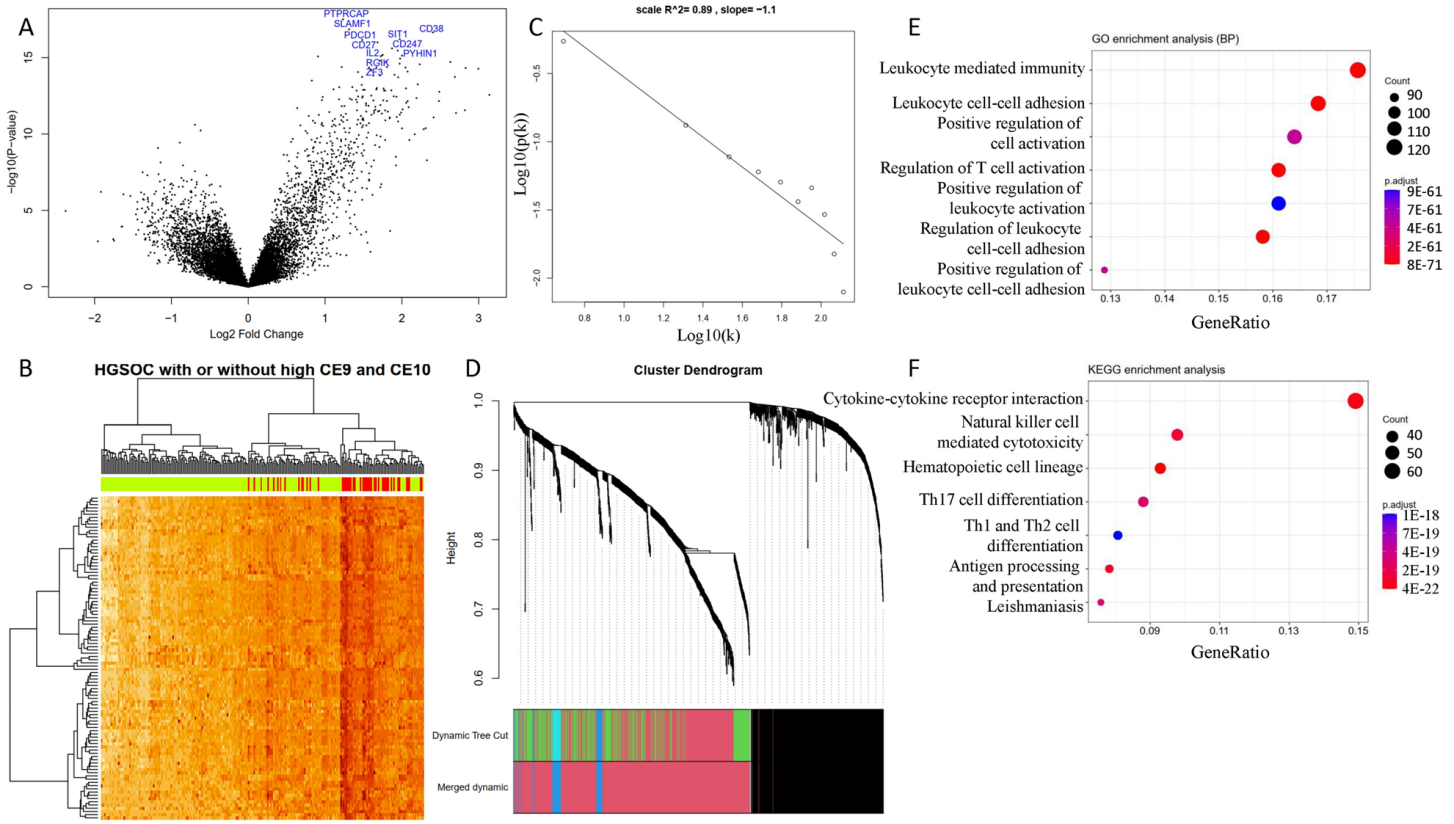
The CE9- or CE10-high expression samples have distinct expression patterns. (**A**) A total of 1265 differential expression genes (DEGs) are identified. (**B**) The clustering heat map based on DEGs shows the distinct expression patterns between groups. In the color bar above the heat map, yellow indicates low CE9- or CE10-low expression samples, while red indicates CE9- or CE10-high expression samples. (**C**) When power was set at 12, the constructed co-expression network based on DEGs followed a power law. (**D**) The cluster dendrogram shows the assignment of genes into gene modules with different colors. The color bar indicates the assignment of genes to a module. (**E**) GO biological process enrichment and (**F**) KEGG enrichment analysis of module M2 genes by clusterProfiler.

As CE9 and CE10 samples had better survival outcomes, we compared the gene expression differences of these patients with others. The differential expression genes (DEGs) were identified by comparing samples assigned to CE9 or CE10 to other samples. A total of 1265 DEGs were identified (Fig. 5A). The 10 most significant genes include *Ptprcap*, *Slamf1*, *Cd38*, *Sit1*, *Pdcd1*, *Cd27*, *Cd247*, *Pyhin1*, *Il2rg*, and *Ikzf3*, all of which were up-regulated in CE9 or CE10 high expression samples. Most of these genes were favorable prognostic factors in an independent dataset (Fig. S2). Cluster analysis based on DEGs showed that CE9 or CE10 high-expression samples were under the same major branch (Fig. 5B).

As these DEGs may be attributed to different types of cells and are involved in distinct biological processes, we performed gene co-expression analysis to check if these genes are organized into modules. Only three modules were identified with distinct biological functions (Fig. 5C,D). M1 was mainly associated with anatomical structure morphogenesis. M2 was associated with adaptive immune response. M4 was associated with innate immune response. Functional enrichment analysis provided more details about M2 functions such as T cell activation, NK cell-mediated cytotoxicity, and Th17 cell differentiation (Fig. 5E,F).

### Ratios of cell state and ecotype are prognostic in HGSOC

**Figure 6:**
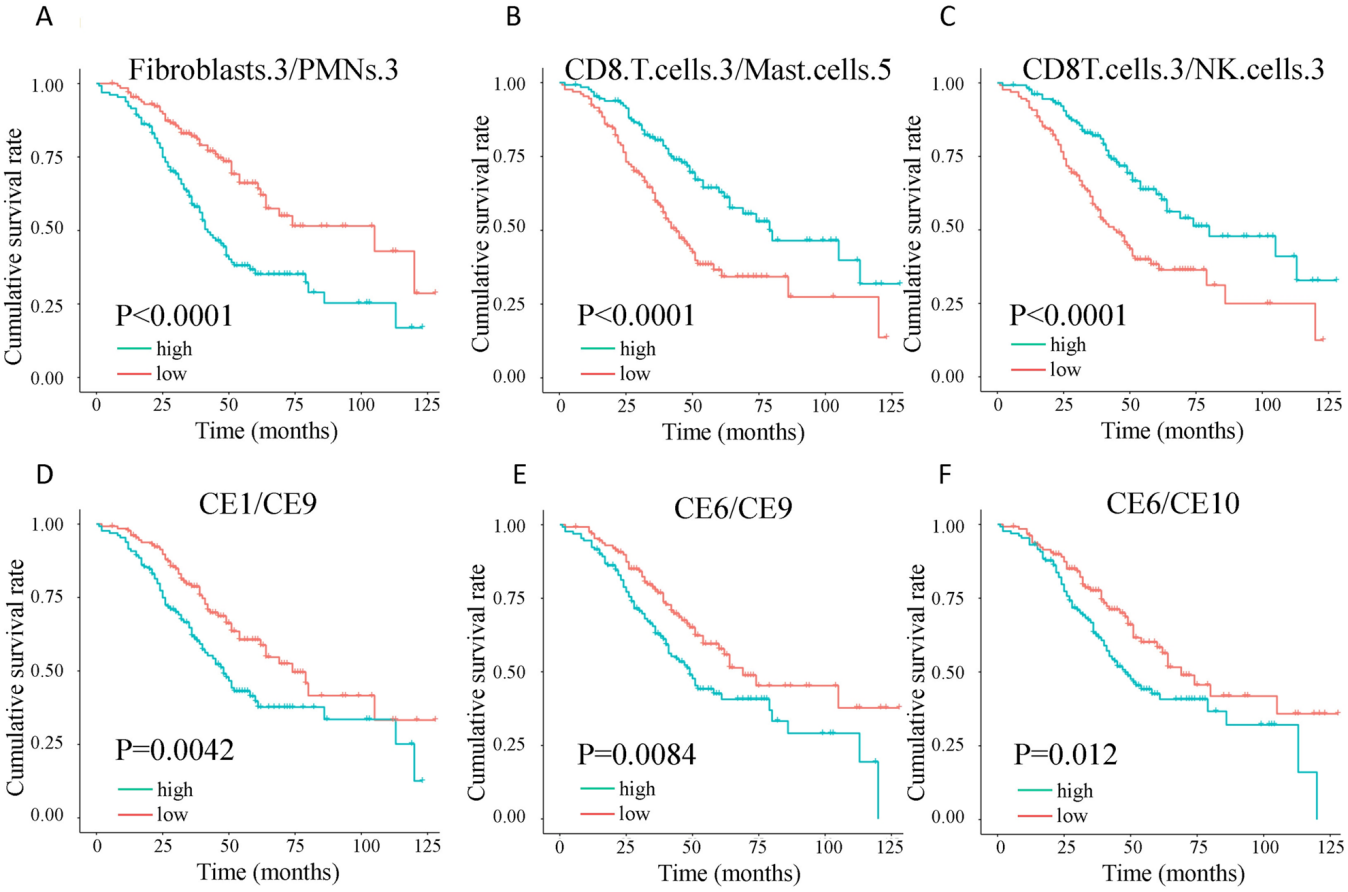
Cell state ratios and ecotypes ratios in HGSOC. Cell state ratios of (**A**) fibroblasts S3/polymorphonuclear leukocytes (PMNs) S3, (**B**) CD8 T cell S3/mast cell S5, and (**C**) CD8 T cell S3/NK cell S3 are associated with HGSOC survival. Ecotype ratios of (**D**) CE1/CE9, (**E**) CE6/CE9, and (**F**) CE6/CE10 are associated with HGSOC survival. The unit of time is the month.

As mentioned previously, the cell state is heterogeneous. We questioned if ratios of different immune cell states could also prognosis. We found hundreds of cell ratios that can significantly separate patients with high and low survival rates. For example, MCs S1/MCs S6 and MCs S3/MCs S6 were both positively associated with survival (Fig. S3A,B). S3 and S6 were annotated as M1 and M2 macrophage subsets, respectively. The three most significant cell state ratios were fibroblast S3/PMNs S3, CD8 T cell S3/mast cell S5, and CD8 T cell S3/NK cell S3 (Fig. 6A–C). The Fibroblast S3 marker gene is *Postn* and the CD8 T cell marker gene is *Ifng*.

At the higher community level, we found that 20 ratios of CE ecotypes were associated with overall survival in HGSOC. The three most significant CE ratios were CE1/CE9, CE6/CE9, and CE6/CE10 (Fig. 6D–F). According to the Ecotyper tool, CE9 and CE10 high-expression tumors were enriched with leukocytes and characterized by higher IFNG signaling and higher B cell content, respectively.

### Potential mechanisms of therapeutic benefit with paclitaxel

**Figure 7:**
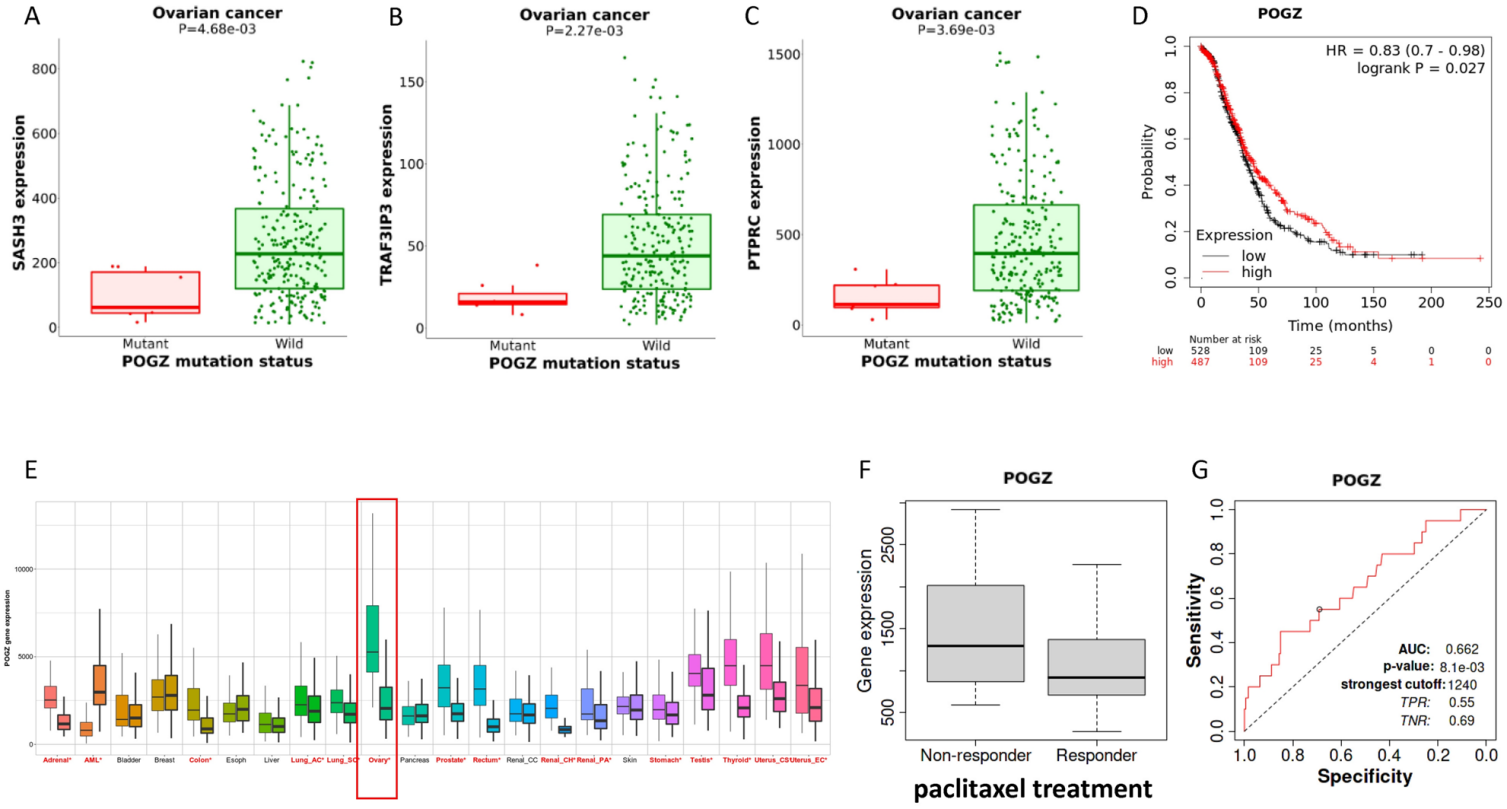
Genome-wide mutation analysis in the TCGA ovarian cancer dataset revealed the association between three module M2 hub genes (**A**) *Sash3*, (**B**) *Traf3ip3*, and (**C**) *Ptprc* and candidate regulatory gene *Pogz*. (**D**) *Pogz* expression is associated with patient overall survival. (**E**) The expression of *Pogz* across 22 different tissues of tumor and normal samples. (**F**) *Pogz* low expression patients response to paclitaxel treatment. (**G**) *Pogz* could separate samples from non-responders and responders to paclitaxel treatment.

We explored the potential of quantitated cell states and cellular ecosystems for predictive biomarker discovery in chemotherapy response. We found that some of the hub genes in co-expression module M2 could be regulated by a common upstream gene P*ogz*. In the TCGA dataset, *Pogz* mutations were associated with elevated genes such as *Sash3*, *Traf3ip3*, and *Ptprc* (Fig. 7A–C), which are marker genes in B cells and macrophages. Survival analysis confirmed that the *Pogz* gene is a protective factor in ovarian cancer (Fig. 7D). In the GTEx dataset, we observed a tissue-specific high expression of *Pogz* in the normal ovary, which is down-regulated in ovarian cancer (Fig. 7E). Thus, we examined *Pogz* expression in an HGSOC dataset, which profiled transcriptome before paclitaxel treatment, and found that *Pogz* low expression was associated with the responder, although the AUC of its predictive ability was moderate (Fig. 7F,G). Further differential analysis showed that *Pogz* altered during the disease progression (Fig. S4A). Cellular expression analysis revealed that *Pogz* was mainly expressed in M2 macrophages (Fig. S4B).

## Discussion

Precise dissection of the tumor tissue transcriptome into cellular composition is critical for understanding tumor heterogeneity and its potential applications in cancer diagnostics and treatment^26^. While state-of-the-art systems biology methods have been developed for human diseases, allowing for in-depth gene-, module- and cellular-level investigation^27^, a comprehensive analysis of HGSOC cell states and ecosystems is lacking. In this study, we utilized the latest Ecotyper method to shed light on the contributions of diverse tumor cell states and ecotypes in HGSOC.

We first used Ecotyper to delineate cell states and ecotypes in HGSOC. Many of these cell states have literature support but were often missed by the traditional CIBSERSORTx analysis^28^. For example, MCs S3 is annotated as an M1 subtype and is associated with longer survival. The favorable roles of M1 macrophages have been reported^3,8,29^. Additionally, DCs S3, characterized as mature immunogenic DCs, are critical for T cell activation^30^. DCs S3 is a favorable factor for survival and its top maker gene is PRF1, whose mutation is associated with hereditary cancer predisposition^31^. Furthermore, some cell states we identified are novel, such as NKs S4 which was associated with unfavorable prognosis. The marker of NKs S4 VAC14 was found to be highly expressed in malignant melanoma but was low in normal skin tissue^32^. While NKs S1 is annotated as classical. The marker of NKs S1 is CD247 which plays an important role in NK cell activation and cytotoxicity^33,34^. In contrast, PCs S1 is a favorable factor for survival and its top maker gene is CD27, whose lowly expression is associated with high-risk disease^35^. CD27, a member of tumor necrosis factor (TNF)-alpha superfamily, is a memory marker of B cells. Lower TNF-alpha significantly induced cell proliferation^36^. Besides, CD27 could promote suppressor activity of immunosuppressive cells such as myeloid-derived suppressor cells (MDSCs) in melanoma^37^. We further explored ECs S4 which is annotated as pro-inflammatory and is a favorable factor for survival. The top marker gene for ECs S4 is *Ltbr*, known for its role in inducing apoptosis in lung epithelial cells^38^. Collectively, our analysis discovered cell subpopulations in HGSOC that may serve as biomarkers and merit further experimental validation.

Our findings are consistent with a recent study that used CIBERSORTx to estimate the abundance of immune cell types from bulk RNA-seq data^16^. They found that activated memory CD4 T cells and plasma cells were associated with improved OS, whereas resting mast cells and M2 macrophages were associated with an increased risk of death. Unsupervised clustering of tumor samples further emphasized the impact of immune cell populations on patient survival. They found patients in cluster IMM.3 (n = 22) had the longest OS, with tumor samples enriched for PCs, activated memory CD4 T cells, M1 macrophages, and resting NK cells. In cutaneous plasmacytomaisease, disease progression was accompanied by a significantly decreased number of NK cells and low NK cell activity in the peripheral blood^39^. Immunostimulatory cytokines such as IL-2 and IL-12 could significantly induce NK cell activity^40^. In our result, CE9 patients have a higher survival rate. Similarly, CE9 is composed of CD4 T cells, M1 macrophages, DCs, and NK cells.

As we quantitated cell states, our investigation extended to the examination of cell state ratios and their prognostic significance. We found that cell ratio exhibits superior predictive abilities with lower P values compared to analyzing by only a single cell state. A notable example is the macrophage M1/M2 ratio, which is also identified in our analysis. A high M1/M2 ratio of tumor-associated macrophage (TAM) has been associated with longer survival in ovarian and breast cancer patients^41,42^. Immunosuppressive IL-10 produced by TAMs could enhance tumor cell survival, proliferation, and metastasis by the inhibition of NK and cytotoxic T cells (CTL) cells^43^. In our analysis, we mathematically identified hundreds of cell state ratios associated with prognosis. However, these ratios need to be biologically annotated by further experimental validation.

Our study also investigated the higher-level tumor ecosystems, revealing the favorable components CE9 and CE10 (Fig. 4), which is consistent with the results from the Ecotype paper^13^. We also found that the ecotype ratios can be prognostic and may result in better predicting performance as the P values are lower than using a single ecotype. We found the three most significant CE ratios were for worse survival (Fig. 6). As CE9 and CE10 are denominators in the ecotype ratios, lower CE9 and CE10 values mean higher ratio values. From a clinical perspective, cell ratios, which are unit-free and potentially comparable across laboratories and experiments, hold promise as practical biomarkers.

To dissect the potential mechanisms of the prognostic ecotype, we conducted traditional DEG analysis to compare CE9, and CE10 samples, and others. Among the 10 most significant DEGs, all genes emerged as favorable predictors for overall survival in an independent dataset, affirming the reliability of Ecotyper. Moreover, we explored potential chemo-resistance mechanisms and identified *Pogz*, an ovary-specific gene, which may regulate some of the differential hub genes in the M2 co-expression module. However, Pogz was down-regulated in the tumor but up-regulated in metastatic cancer. We found that Pogz was mainly expressed in M2 macrophages. M2 macrophages are promoters of metastasis and are associated with poor outcome^9^. Therefore, elevated *Pogz* expression was observed in metastatic cancer (Fig. S4). In sum, *Pogz* is associated with disease progression and paclitaxel treatment response.

Finally, our findings offer valuable insights into potential biomarkers that could aid in identifying HGSOC patients likely to respond to treatment. Employing digital pathology and computational analysis tools that calculate the density of intraepithelial CD8^+^ tumor-infiltrating lymphocytes (TILs) across tumor tissues, holds promise for improving the identification of long-term survivors and predicting the likelihood of positive responses to immune checkpoint blockade^44,45^. Categorizing solid tumors into distinct groups, such as T cell-inflamed, excluded, and non-inflamed, opens avenues for tailoring therapeutic strategies based on the specific tumor microenvironment^44^. In theory, T cell-inflamed HGSOC tumors are promising candidates for immune checkpoint blockade, as their tumor microenvironment is already favorable for an immune attack. For instance, Ecotyper can be used to calculate the density of intraepithelial MCs, then CSF-1R blockade with PLX3397 can be applied to inhibit M2 macrophage adhesion and reprograms M2 macrophages to M1, which may prolong patient survival^46^. Therefore, future research could further explore the impact of different cell states on tumor biology using digital pathology techniques.

## Conclusions

This is the first study to demonstrate the clinical relevance of cell states, ecosystems, and their ratios in HGSOC. The low expression of the *Pogz* gene is associated with paclitaxel treatment responder. The results may expand our understanding of the cellular organization in HGSOC with implications for disease mechanisms, disease diagnosis, and precision therapies.

### Supplementary Information

Supplementary Figures.

Supplementary Tables.

## Data Availability

The dataset analyzed in the study is available at the public database NCBI GEO (https://www.ncbi.nlm.nih.gov/geo/).
