# Supplementary material for: Immune cell landscapes are associated with high-grade serous ovarian cancer survival
Source: Sci Rep. 2024 Jul 12;14:16140. doi: 10.1038/s41598-024-67213-4 (PMC11245545; doi:10.1038/s41598-024-67213-4)
Supplement: Supplementary file 1 — Supplementary Figures. [file 41598_2024_67213_MOESM1_ESM.docx]

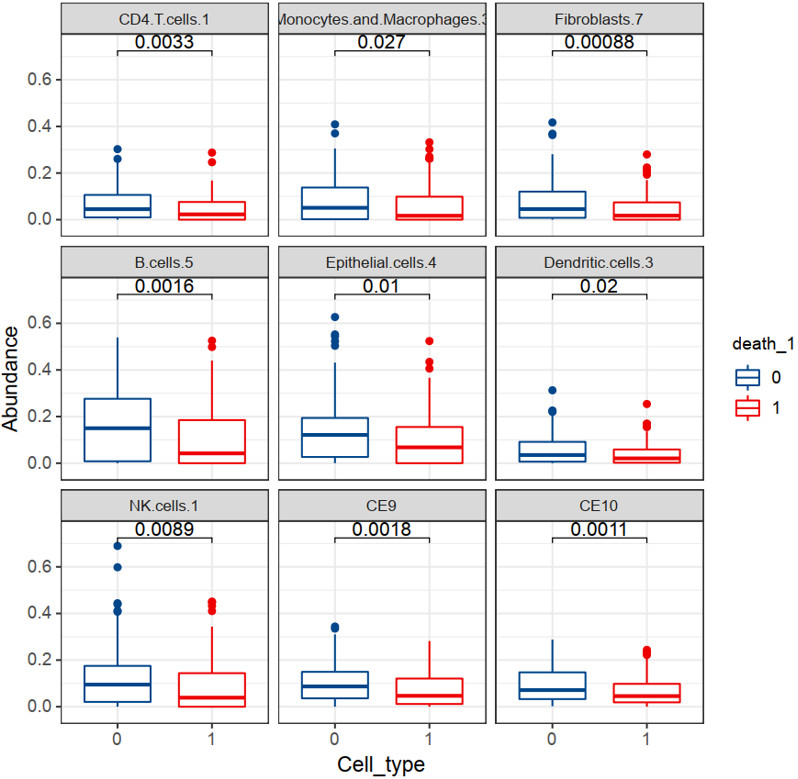


**Figure S1** Box plots showing the differential expression of nine cell states between died and survived HGSOC patients. 1: died, 0: survived.


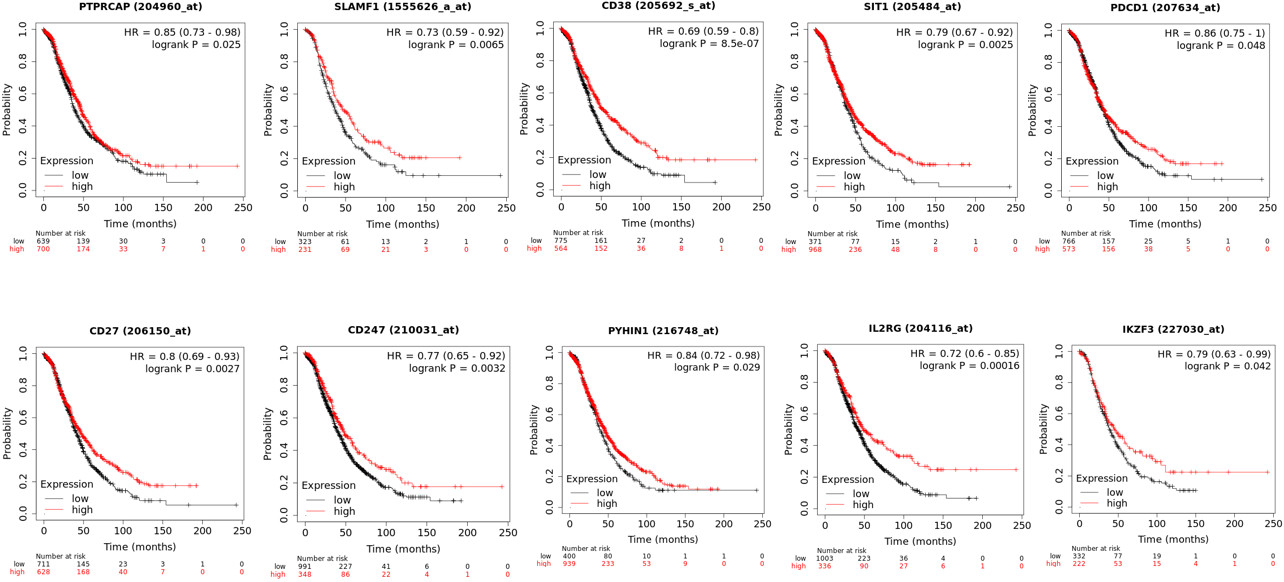


**Figure S2** The top 10 most significant DEGs *Ptprcap*, *Slamf1*, *Cd38*, *Sit1*, *Pdcd1*, *Cd27*, *Cd247*, *Pyhin1*, *Il2rg*, and *Ikzf3* are all predictive of ovarian cancer overall survival in an independent dataset in Kaplan-Meier Plotter database.


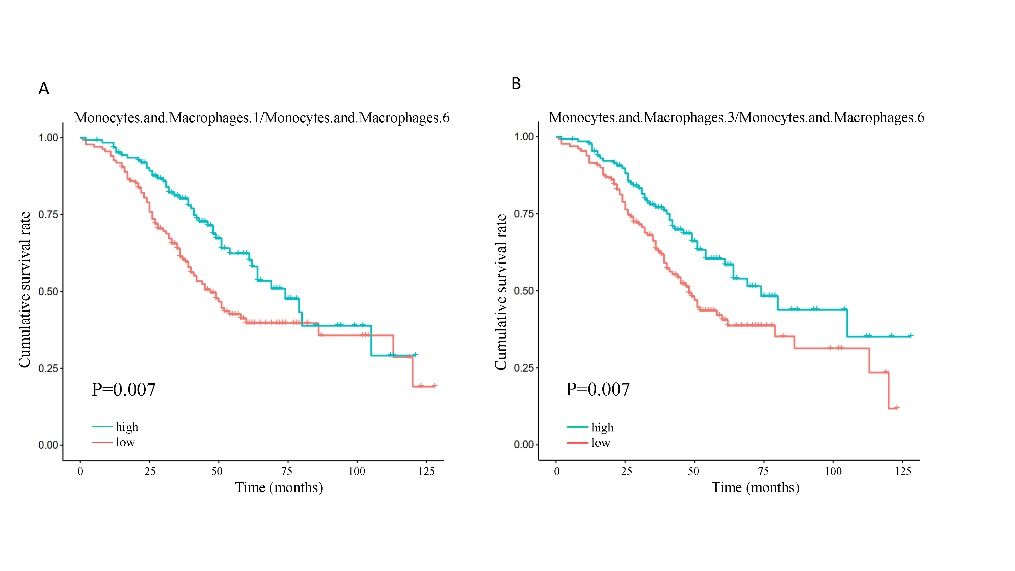


**Figure S3** Cell state ratios of (A) macrophages (MCs) S1/S6 and (B) MCs S3/S6 are predictive of HGSOC survival.


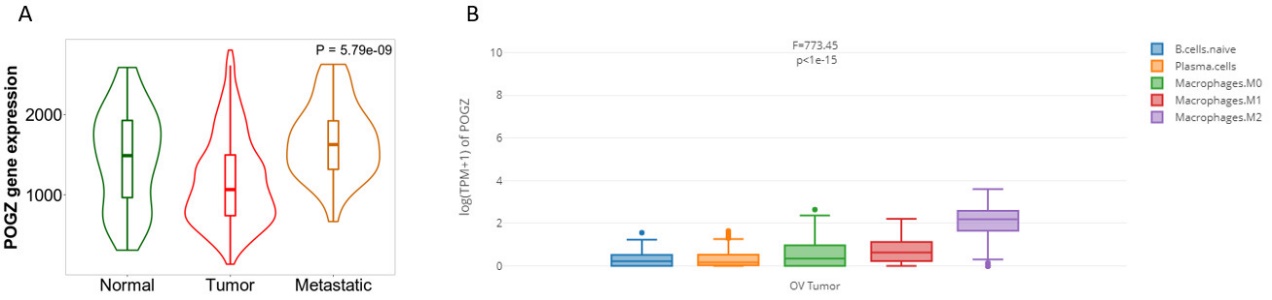


**Figure S4** *Pogz* expression in ovarian cancer. (B) *Pogz* is differentially expressed in TCGA ovarian cancer dataset during the disease development. *Pogz* shows significant differential expression between macrophages M2 and other tumor microenvironment cells.
